# Veno-Arterial-Venous Extracorporeal Membrane Oxygenation in a Patient Undergoing Dialysis and Having Cardiopulmonary Failure Due to Coronavirus Disease: A Case Report

**DOI:** 10.7759/cureus.69317

**Published:** 2024-09-13

**Authors:** Hideya Itagaki, Yuuto Motoyoshi, Misako Nagai, Yoshinobu Abe, Nobutoshi Matsumura, Tomoyuki Endo

**Affiliations:** 1 Emergency and Disaster Medicine, Tohoku Medical and Pharmaceutical University Hospital, Sendai, JPN

**Keywords:** cardiogenic shock, cardiomyopathy, covid-19, dialysis, extracorporeal membrane oxygenation, renal failure

## Abstract

Coronavirus disease (COVID-19), a viral infection caused by severe acute respiratory syndrome coronavirus 2, was first reported in China in December 2019 and has since become a global pandemic. COVID-19 is a multisystem disease with respiratory symptoms as the main presentation. There is growing awareness of the adverse prognostic impact of cardiovascular involvement caused by COVID-19. We report a case of a patient on hemodialysis with COVID-19 who developed cardiopulmonary failure and was successfully weaned off veno-arterial-venous (VAV) extracorporeal membrane oxygenation (ECMO).

A 54-year-old man was brought to our intensive care unit (ICU) with respiratory failure due to COVID-19, which had been diagnosed based on antigen testing results. Three days prior, he had started taking dexamethasone orally, but his respiratory distress had worsened two days prior, and he was referred to our hospital. He had a history of hypertension, atrial fibrillation, chronic heart failure, and end-stage renal disease. He was found to have circulatory shock and severe hypoxemia, and computed tomography (CT) showed ground-glass opacities throughout the lung fields. Hypoxemia persisted after ventilation. The ventilation settings for this patient were volume control ventilation, fraction of inspiratory oxygen 100%, tidal volume 360 ml, respiratory rate 22 breaths per minute, and positive end-expiratory pressure 15 mmHg. Therefore, veno-venous ECMO (VV-ECMO) was initiated. Echocardiography showed right ventricular free-wall motion disorder, which was judged to be a complication of cardiogenic shock due to septic cardiomyopathy. The patient was switched to VAV-ECMO, after which his circulatory and respiratory insufficiency gradually improved. He was switched to VV-ECMO on day 7 of hospitalization and weaned off ECMO on day 15 of hospitalization. The patient was fully weaned off the ventilator on day 37 of hospitalization and discharged from the ICU on day 38 of hospitalization.

COVID-19 can cause cardiomyopathy, a rare cardiovascular disorder that can lead to cardiogenic shock; however, the cardiac and pulmonary symptoms may not occur simultaneously. Therefore, switching to VAV-ECMO after VV-ECMO was introduced; however, it is important to note that the mortality rate associated with this procedure is high.

## Introduction

Coronavirus disease 2019 (COVID-19), a viral infection caused by severe acute respiratory syndrome coronavirus 2, was first reported in Wuhan, China, in December 2019 and has since become a global pandemic [1]. COVID-19 is a multi-organ disease that mainly has respiratory symptoms and often causes respiratory failure. Veno-venous (VV) extracorporeal membrane oxygenation (ECMO) has improved survival rates in patients with COVID-19. COVID-19 can also cause cardiovascular complications and, in rare cases, cardiogenic shock, for which veno-arterial (VA) ECMO and veno-arterial-venous (VAV) ECMO are occasionally used [2,3]. Myocardial injury is estimated to occur in up to 20-30% of COVID-19 patients, and in severe cases, cardiopulmonary failure may result [4]. It is estimated that VA-ECMO or VAV-ECMO is performed in 4-8% of patients with COVID-19 cardiopulmonary failure [4,5]. However, the mortality rate of patients with COVID-19 who receive VA-ECMO or VAV-ECMO is high, and end-stage renal failure with the induction of dialysis, as in the present patient, is a risk factor for high mortality [4-6]. Herein, we present a case of a patient on dialysis with COVID-19 who had cardiopulmonary failure and was successfully weaned off dialysis after VAV-ECMO.

## Case presentation

A 54-year-old Japanese man was brought to our intensive care unit (ICU) with severe respiratory failure due to COVID-19. He had a fever for five days before visiting our hospital, and four days prior to his arrival, he visited a nearby hospital and was diagnosed with COVID-19 by antigen testing. He was started on oral dexamethasone; however, his respiratory distress gradually increased. Immediately before his arrival, the patient was seen again at the nearby hospital and was transported to our hospital owing to hypoxemia. He had a history of hypertension, atrial fibrillation, chronic heart failure, and end-stage renal disease owing to hereditary nephropathy and had been on hemodialysis thrice a week for >10 years. The patient also received evocalcet, esomeprazole, vericiguat, lanthanum carbonate hydrate, ferric citrate hydrate, and precipitated calcium carbonate and had received three doses of the coronavirus vaccine. His smoking history was 20 cigarettes per day from the age of 20 to 45 years; however, he had not smoked recently.

The patient was 166 cm tall, weighed 89 kg, and had a body mass index of 32 kg/m^2^. Vital signs included a blood pressure of 74/32 mmHg, pulse rate of 149 bpm, oxygen saturation (SpO_2_) of 85% (on 15 L oxygen), respiratory rate of 35-50 times/second, and body temperature of 36.0 ºC with clammy extremities; orthopnea and wheezing respiratory sounds were noted. Blood tests revealed an elevated inflammatory response, coagulopathy, elevated markers of myocardial injury, and renal dysfunction (Table 1). 

**Table 1:** Laboratory findings at the time of admission Alb: Albumin; ALP: Alkaline phosphatase: ALT: Alanine aminotransferase; APTT: Activated partial thromboplastin time; AST: Aspartate aminotransferase; BUN: Blood urea nitrogen; Ca: Calcium; CK: Creatine kinase; CK-MB: Creatine kinase-myoglobin binding; Cl: Chlorine; Cre: Creatinine; CRP: C-reactive protein; γGT: γ-glutamyl transpeptidase; Hb: Hemoglobin; Hct: Hematocrit; IL-6: Interleukin-6; K: Potassium; LDH: Lactate dehydrogenase; Mg: Magnesium; Na: Sodium; Plat: Platelet; PT: Prothrombin time; PT-INR: Prothrombin time-international normalized ratio; PCT: Procalcitonin; RBC: Red blood cell; T-bil: Total bilirubin; TP: Total protein; Troponin T: Cardiac muscle troponin T; WBC: White blood cell; Fib: Fibrinogen; MCH: Mean corpuscular hemoglobin; MCV: Mean corpuscular volume; Lac: Lactate; HCO^3-^: Bicarbonate; PO_2_: Partial pressure of oxygen; PCO_2_: Partial pressure of carbon dioxide

Parameters	Patient Values	Units
Complete Blood Count Data		
WBC	19	10^3^/μL
RBC	5	10^6^/μL
Hb	15.8	g/dL
Hct	48.1	%
Plat	131	10^3^/μL
MCV	96.1	fL
MCH	31.5	pg
Fib	919	mg/dL
APTT	46.5	sec
PT-INR	1.15	
D-dimer	7.57	μg/mL
Biochemistry Data		
TP	6.8	g/dL
Alb	3.1	g/dL
T-bil	1.11	mg/dL
AST	60	U/L
ALT	40	U/L
ALP	68	U/L
γGT	52	U/L
LDH	569	U/L
BUN	45	mg/dL
Cre	10.17	mg/dL
Na	141	mmol/L
K	4.6	mmol/L
Cl	93	mmol/L
Ca	8.4	mg/dL
CK	109	U/L
CK-MB	26	U/L
Tropnin T	0.052	ng/mL
CRP	42.47	mg/dL
PCT	5.75	ng/mL
Blood Gas Analysis		
pH	7.268	
PCO_2_	38.5	mmHg
PO_2_	60.3	mmHg
HCO^3-^	17	mmol/L
Lac	7.5	mmol/L

**Figure 1:**
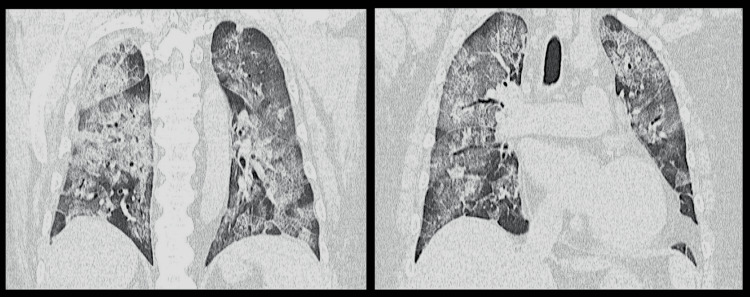
CT scan showing ground-glass shadows throughout both lung fields CT: Computed tomography

Echocardiography showed an ejection fraction of more than 50%, an inferior vena cava diameter of more than 20 mm, and a peak tricuspid regurgitation gradient of 39 mmHg, suggesting right ventricular free wall motion impairment. Computed tomography (CT) showed ground-glass opacity over the entire lung field (Figure 1), and the patient was immediately placed on a ventilator.

The ventilator settings were: 100% inspiratory oxygen concentration (FiO2), 360 mL of ventilation, and 10 cm H_2_O of positive end-expiratory pressure. The arterial blood gas analysis showed pH 7.187, arterial PCO_2_ (PaCO_2_) 53.9 mmHg, and arterial PO_2_ (PaO_2_) 69.2 mmHg (Table 1). 

**Figure 2:**
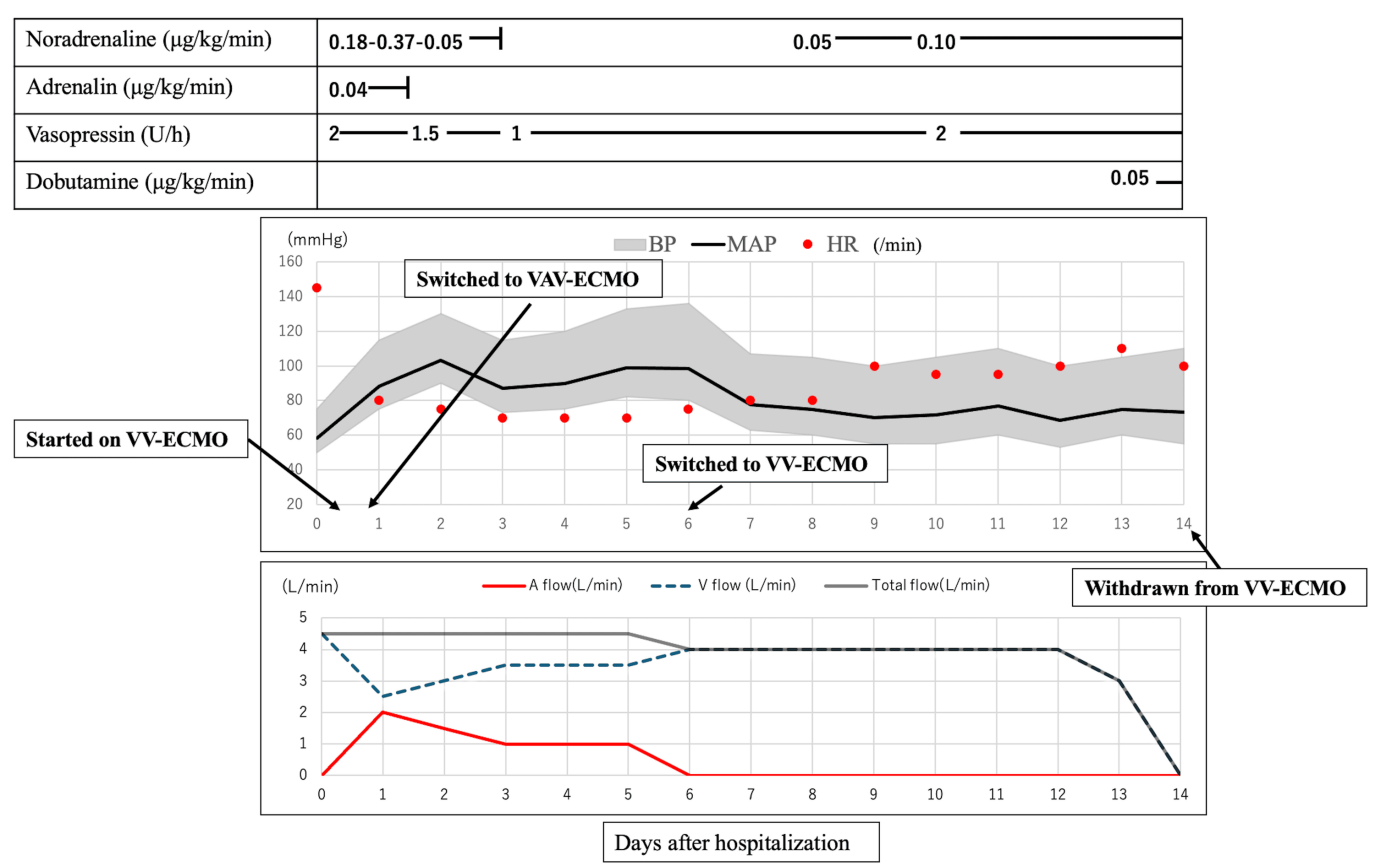
Time course of COVID-19 The bottom figure shows the A-flow and V-flow processes. A-flow: Flow rate to arterial side; V-flow: Flow rate to venous side; VAV-ECMO: Veno-arterial-venous extracorporeal membrane oxygenation; VV-ECMO: Veno-venous extracorporeal membrane oxygenation; COVID-19: Coronavirus disease; BP: Blood pressure; HR: Heart rate; MAP: Mean arterial pressure

After changing his end-expiratory positive pressure from 10 cm H_2_O to 15 cm H_2_O, he continued to have poor oxygenation; therefore, VV-ECMO was introduced. A 20 Fr cannula was inserted into the right internal jugular vein and a 25 Fr cannula into the right femoral vein, and the patient underwent pumping for three hours and 30 minutes after admission to the ICU (Figure 2). 

After the introduction of VV-ECMO, the patient continued to develop hypotension despite the use of hypertensive drugs (noradrenaline, vasopressin, and adrenaline). When echocardiography was repeated, the patient developed right ventricular free-wall motion disorder, which was complicated by cardiogenic shock from septic cardiomyopathy (Video 1).

**Video 1:** Echocardiogram before introduction of VAV-ECMO Echocardiography showing global wall motion loss in the left ventricle and wall motion loss in the right ventricle. VAV-ECMO: Veno-arterial-venous extracorporeal membrane oxygenation

A 16 Fr blood cannula was inserted into the right femoral artery, and the patient was switched from VV-ECMO to VAV-ECMO five hours later (Figure 2). The flow rate was controlled using an occluder; the flow on the arterial side was 2 L/min and the settings were: flow 4.5 L/min (A: 2.0 L/min; V: 2.5L/min); FiO_2_ 100%; gas flow 6 L/min; and venous oxygen saturation (SvO_2_) 75% (Figure 2). Dialysis was also initiated at the time of ECMO introduction in the form of continuous hemodialysis and filtration, with initial blood flow (QB) settings of 60 mL/min, dialysate flow of 300 mL/min, and replacement fluid flow of 300 mL/min. 

After VAV-ECMO was performed, the patient’s blood pressure improved, and the blood flow was set at 80 mL/min. The circulatory and respiratory insufficiency gradually improved, and the ultrafiltration rate was initiated at 100 mL/h. The patient was transferred to VV-ECMO on day 7 of hospitalization and was withdrawn from ECMO on day 15. He was weaned from ventilation on day 37 of hospitalization and was discharged from the ICU on day 38 of hospitalization (Figure 2). After discharge, the patient continued dialysis management and rehabilitation on a general bed and survived for more than 90 days after hospitalization.

## Discussion

We report the case of a patient on dialysis who experienced cardiogenic shock from septic cardiomyopathy caused by COVID-19, which led to the introduction of VAV-ECMO. To our knowledge, this is the first case of a patient with COVID-19 with end-stage renal failure who required dialysis, was weaned off VAV-ECMO, and survived for more than 90 days.

COVID-19 causes a variety of complications but rarely cardiovascular damage. It has been suggested that direct infection of the myocardium with COVID-19 may cause myocardial cell death, and inflammation from cytokine storms may directly cause myocardial damage; however, the underlying mechanisms remain unclear [2]. Previous studies have reported an association between acute myocarditis and COVID-19. Acute myocarditis occurred in 2.4 patients per 1,000 admissions, 38.9% of whom had fulminant disease and required inotropic agents or temporary mechanical assistance [2]. In our case, the myocardial motion deteriorated rapidly from the time of admission, and both ventricles became sluggish. Considering that the myocardial motion subsequently improved, septic cardiomyopathy was the most likely cause.

However, including our patient, VA-ECMO is required in 3-4% of patients with COVID-19 who require ECMO, while VAV-ECMO is required in approximately 1% of patients. Furthermore, among the 652 patients in the Extracorporeal Membrane Oxygenation for Respiratory Failure and/or Heart failure related to Severe Acute Respiratory Syndrome-Coronavirus 2 (ECMOSARS) registry, only seven were converted from VV-ECMO to VAV-ECMO [5,7,8]. Although the probability is approximately 1%, the median time between the onset of COVID-19 symptoms and the appearance of cardiac symptoms is five days; thus, it is necessary to assume that a certain number of patients will change from VV-ECMO to VAV-ECMO, as did our patient [2]. 

The in-hospital mortality of patients treated with VAV-ECMO is high, and, in general, the in-hospital mortality rate for patients with COVID-19 treated with VV-ECMO is estimated to be approximately 40%. The mortality rate of patients with COVID-19 treated with VA-ECMO or VAV-ECMO is even higher, and a study in the ECMOSARS registry of patients with COVID-19 treated with VA-ECMO or VAV-ECMO reported an in-hospital survival rate of 28% (survivors were followed up for 90 days) [5,7,9]. Another study reported a 64% mortality rate for patients with COVID-19 who required VA-ECMO or VAV-ECMO, along with an extremely high mortality rate (82%) for patients who switched from VV-ECMO to VA-ECMO or VAV-ECMO [4]. The mortality rate of patients who switched from VAV-ECMO to VV-ECMO was moderately lower, at 57% [4].

In addition to the severe prognosis of VAV-ECMO in patients with COVID-19, our patient was also at risk of end-stage renal failure and required dialysis. Patients having end-stage renal failure with chronic renal failure or who are on dialysis tend to have worse clinical outcomes because of their marked vulnerability to COVID-19, with mortality rates as high as 19.4% among patients admitted with COVID-19 [6,10]. Considering this situation, the conversion of patients with end-stage renal failure who require dialysis with VAV-ECMO insertion, as in our patient, is expected to be quite challenging. However, currently, there is insufficient information available to consider the use of VA-ECMO or VAV-ECMO in patients on dialysis. Among the patients with COVID-19 in the ECMOSARS registry mentioned previously, only 11 (4%) had chronic renal failure, of whom only four with chronic renal failure used VA-ECMO or VAV-ECMO [5]. Notably, only four of these 11 patients survived, and all of those who received VA-ECMO or VAV-ECMO died [5]; therefore, the indications for ECMO, especially VA-ECMO or VAV-ECMO, in patients undergoing dialysis should be carefully evaluated. Clinicians also need to understand that VAV-ECMO can provide a viable solution to save a patient's life because, as in this patient, patients on dialysis who receive VAV-ECMO can survive beyond 90 days.

## Conclusions

In summary, COVID-19 can cause cardiomyopathy, a rare cardiovascular disorder that can lead to cardiogenic shock. The cardiac and respiratory symptoms may not occur simultaneously, and it is possible to switch to VAV-ECMO after the induction of VV-ECMO; however, it is important to note that the mortality rate is high. In addition, patients on dialysis are at risk of worse clinical outcomes owing to COVID-19, and no reports of adequate survival in patients on dialysis who have received VAV-ECMO have been reported. It is important for clinicians to understand that, with careful case evaluation, VAV-ECMO can be an option to save patients, as in the case reported herein.
